# The Effect of the Application of Chemical Fertilizer and Arbuscular MyCorrhizal Fungi on Maize Yield and Soil Microbiota in Saline Agricultural Soil

**DOI:** 10.3390/jof11040319

**Published:** 2025-04-17

**Authors:** Ye Yuan, Zhengjun Feng, Shengxin Yan, Junjie Zhang, Huiping Song, Yan Zou, Dapeng Jin

**Affiliations:** 1 Institute of Resources and Environmental Engineering, Shanxi University, Taiyuan 030006, China; yeyuan7814@126.com (Y.Y.); afattt@163.com (S.Y.); 13466840125@163.com (J.Z.); songhp@sxu.edu.cn (H.S.); 2 Engineering Research Center of Resource Efficiency Enhancing and Carbon Emission Reduction in Yellow River Basin, Ministry of Education of the People’s Republic of China, Taiyuan 030006, China; 3 Shanxi Yellow River Laboratory, Taiyuan 030006, China; 4 Shanxi Qinghuan Nengchuang Environmental Protection Technology Co., Ltd., Taiyuan 030006, China; 202214001013@email.sxu.edu.cn (Y.Z.); 18303461074@163.com (D.J.)

**Keywords:** chemical fertilizer, arbuscular mycorrhizal fungi (AMF), saline–sodic soil, soil microbial community

## Abstract

The overuse of chemical fertilizers not only leads to resource wastage but also causes problems such as environmental pollution and soil degradation. In particular, crop growth in saline–sodic soils is severely restricted due to high salinity and alkalinity, further exacerbating challenges in agricultural production. The aim of this study was to investigate different fertilization strategies that combine chemical fertilizer reduction with arbuscular mycorrhizal fungi (AMF) for improving saline–sodic soils and to assess the effects of these protocols on crop yield, soil properties, and microbial communities. Field experiments across two sites (BeiWuLao and XuJiaZhen) demonstrated that integrating AMF with CF reduction (AHCF treatment) significantly enhanced maize yield by 23.5% at BeiWuLao (from 11,475 to 14,175 kg/ha) and 81.2% at XuJiaZhen (from 7245 to 13,125 kg/ha) compared to conventional fertilization (CK) (*p* < 0.01). Soil nutrient analysis revealed substantial improvements: available potassium (AK) increased by 77.7% (61.35 vs. 39.33 mg/kg), available phosphorus (AP) by 33.9% (20.50 vs. 15.50 mg/kg), ammonium nitrogen (AN) by 57.3% (64.17 vs. 40.83 mg/kg), and soil organic matter (SOM) by 96.4% (46.98 vs. 23.91 mg/kg) under AHCF treatment (*p* < 0.05). Although pH and electrical conductivity (ECe) remained unaffected, AMF inoculation shifted microbial composition, elevating salinity-tolerant taxa such as Actinobacteria (+24.7%) and Anabaena. Beta diversity analysis (PCoA) confirmed distinct microbial community structures between treatments, with ECe and AN identified as primary drivers of bacterial (RDA variance: 74.08%) and fungal (RDA variance: 54.63%) communities, respectively. Overall, the combination of chemical fertilizer reduction and AMF effectively improved soil fertility, microbial community structure, and crop yield. These findings have important implications for improving saline soils and promoting environmental sustainability.

## 1. Introduction

Saline–alkali lands, found in over one hundred countries, cover an extensive area of approximately 955 million hectares, accounting for approximately 25% of the Earth’s total land surface [1]. In China, saline–sodic soils are primarily located in the northeastern, northern, and northwestern inland regions. The total area of saline–alkali soils in the country is approximately 33 million hectares, representing approximately 4.88% of the total land area, with an annual increase of 1% [2]. Excessive accumulation of sodium and elevated soil pH adversely impact soil structure and hydraulic properties, leading to a reduction in crop yields [3,4]. In arid and semi-arid regions, insufficient precipitation, high temperatures, increased evaporation, and inadequate farmland management further exacerbate these challenges.

Numerous methods have been developed to reduce soil sodium content, lower soil pH, and enhance the properties of saline–alkali soils [5,6,7]. Although the short-term improvements in soil quality from these methods can be significant, the economic costs are often high, and sustaining these improvements over the long term can be challenging, particularly when saline–alkali conditions are influenced by natural environmental factors [8]. Cultivating saline–alkali-tolerant plants or enhancing the tolerance of existing plants in saline–alkali lands is a more cost-effective and sustainable approach to addressing this issue. Mycorrhizal fungi are a key component of soil diversity in most terrestrial ecosystems. Among the various types of mycorrhizae that form symbiotic relationships with plants, arbuscular mycorrhizal fungi (AMF) are the most prevalent, colonizing root systems [9]. Researchers have found that arbuscular mycorrhizal fungi (AMF) can enhance the saline–alkali tolerance and potential productivity of host plants [10,11,12,13]. Consequently, a thorough investigation into the application of arbuscular mycorrhizal fungi (AMF) in saline–alkali soils is of significant importance.

Chemical fertilizers, a common strategy in global agricultural management, not only enhance crop productivity but also improve the ecological health of soil environments [14]. In the Loess Plateau of China, fertilizer application rates have reached as high as 225–330 kg ha^−1^, significantly exceeding international standards [15]. The overuse of chemical fertilizers exacerbates several cultivation challenges, including soil acidification, salinization, and nutrient imbalances [16,17]. Therefore, there is an urgent need to reduce agricultural consumption of chemical fertilizers in China. However, concerns about potential yield reductions from this reduction pose significant barriers for farmers. Arbuscular mycorrhizal fungi (AMF) can enhance plant uptake of soil nutrients through extensive mycelial and mycorrhizal networks, thereby improving fertilizer utilization efficiency [18]. Combining AMF application with the reduction of chemical fertilizers appears to be a viable solution to this dilemma. AMF can enhance nutrient absorption in plants, helping to counteract potential deficiencies after fertilizer reduction and maintain crop yields at existing levels.

Soil microbial diversity is a valuable indicator of soil quality, reflecting sensitivity to changes in soil nutrients, pH levels, organic matter content, and other factors [19,20,21]. The application of chemical fertilizers significantly affects the microbial community in saline soils [22,23]. Additionally, some researchers have investigated the effects of AMF on microbial communities in these soils [24,25,26]. However, there is a lack of research on how different fertilization regimes affect AMF-induced shifts in soil microbial communities, and the interplay between AMF and chemical fertilizers remains underexplored. This study aimed to investigate the effects of combining reduced chemical fertilizer application with AMF on soil properties and microbial community composition in saline soils.

## 2. Materials and Methods

The research focused on two saline farmlands in Wenshui County, Shanxi Province, which have been affected by historical factors and excessive fertilization. By applying chemical fertilizers (CF) alongside microbial inoculants primarily composed of AMF, the study evaluated improvements in saline–alkali lands and crop growth under various reduced fertilization regimes. Key aspects assessed included crop yield, soil properties, and changes in microbial communities. Additionally, the research explored and demonstrated the efficacy of different formula combinations in enhancing fertilizer utilization efficiency and improving the ecological quality of the land.

### 2.1. Experimental Sites and Study Design

In this investigation, field experiments were conducted at two distinct locations: XuJiaZhen (Xu) Village (112.14° E, 37.28° N) and BeiWuLao (Bei) Village (112.03° E, 37.37° N), both located in Wenshui County, Luliang City, Shanxi Province. The selected sites experience a warm-temperate semi-humid continental climate at an elevation of 1807 m above sea level. They have an average annual temperature ranging from 9 to 12 °C, with approximately 2551.9 h of sunshine per year, precipitation levels between 450 and 700 mm, and a frost-free period of up to 183 days. This climate is essential for the successful growth and maturation of maize crops. The physical and chemical characteristics of the local soil are detailed in Table 1 below.

**Figure 1:**
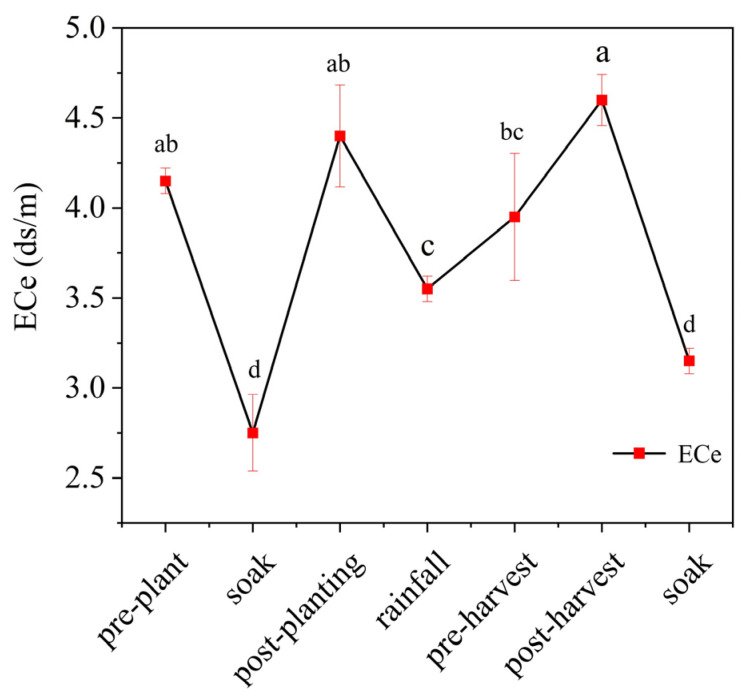
Trend of water salinity dynamics in the experimental field. Bars represent the mean ± SE (*n* = 5 biological replicates). Error bars denote the standard error of the mean. Bars sharing the same lowercase letter are not significantly different (Tukey’s HSD test, *p* < 0.05). Letters assigned based on post hoc comparisons, with alphabetical order unrelated to treatment effect magnitude.

Over the years, leaching has been used locally to improve salinized areas. At the same time, large amounts of cattle manure are applied annually to agricultural lands as a byproduct of the local farming industry, which exacerbates the salinization of agricultural soils. To gain deeper insights into the dynamics of local soil water and salt, the soil electrical conductivity (ECe) at the experimental site was meticulously monitored every other month. As a result, a comprehensive graph was created to illustrate the temporal trends and variations in soil ECe values related to different planting stages over the course of a year, as shown in Figure 1 below:

Through field research, sampling, and analysis, it has been determined that the ECe values of saline–alkali lands in the local area respond to variations in water content within farmland, as illustrated in Figure 1. Before annual fertilization, farmers employ a method called “soaking the land with large volumes of water” to reduce the electrical conductivity of the topsoil. After fertilization, as ions from organic and chemical fertilizers are released, the soil’s free electron concentration increases, causing the ECe value to gradually rise.

During the summer months, characterized by increased precipitation, some soil ions are washed away by rainfall, leading to a further decrease in ECe values. However, in certain low-lying plots, drainage issues can cause salt accumulation, resulting in higher ECe values. Following crop harvest, nutrients from excessive fertilization that were not utilized continue to release salt ions, further concentrating in the soil and causing the ECe value to rise. Consequently, during the winter months, the practice of “soaking the land with large volumes of water” is continued to lower the ECe values in the farmland. This sequence of events reveals the regular pattern of water and salt dynamics observed in the experimental fields.

The experiment was carried out on 28 April 2023 in two typical saline farmlands in Wenshui County, Shanxi Province—BeiWuLao and XuJiaZhen, which differed in the initial physicochemical properties of the soils (Table 1), but were treated with the same fertilizer treatment regimen in order to compare the regional differences in response. Five fertilizer treatments were set up in the experiment, and each treatment was independently set up in three biological replications in both sites, with a total of 30 plots (2 sites × 5 treatments × 3 replications), with a single plot area of 20 m^2^, and a 1 m isolation zone was set up between the plots to avoid cross-pollution. The three replications of each treatment within a single site met the requirements of analysis of variance (ANOVA) (α = 0.05), and the differences between groups were compared by Duncan’s post hoc test to ensure that the experimental data could effectively reflect the treatment effects. Differences in initial soil properties (pH, conductivity, organic matter and nutrient content) between the two sites are detailed in Table 1 to provide basic data for subsequent analysis of regional response differences. The specific fertilizer management for each group is as follows: (1) CK: conventional fertilization; (2) CF: chemical fertilizer at 600 kg/ha; (3) HCF: chemical fertilizer at 300 kg/ha; (4) ACF: chemical fertilizer at 600 kg/ha and AMF at 157.5 kg/ha; (5) AHCF: chemical fertilizer at 300 kg/ha and AMF at 157.5 kg/ha.

Utilizing the quintile sampling methodology [27], we procured a 200 g soil sample, which was thoroughly mixed using the quartering technique. Soil was collected from depths ranging from 0 to 20 cm. After grinding and sieving the samples (<2 mm), each was air-dried for subsequent chemical analysis.

### 2.2. Makings

Arbuscular mycorrhizal fungi (AMF) were obtained from Norman Environmental Technologies, Czech Republic, with a spore density of (80 ± 5) spores/g and a percentage of active spores greater than 90%. Fertilizers were purchased from Hubei Chufeng Fertilizer Trading Co. Ltd. in Guangzhou, China, including urea (N 46%), calcium superphosphate (P_2_O_5_ 18%), and potassium sulfate (K_2_O 50%), and were mechanically deep-applied 7 days before planting (15–20 cm soil layer). The maize (*Zea mays* L.) variety used in the experiment was “Xianyu 1321”, purchased from the Chinese Academy of Agricultural Sciences (Beijing, China).

### 2.3. Methods

#### 2.3.1. Analysis of Soil Physical and Chemical Properties

The soil was leached at a water to soil ratio of 1:2.5 and the pH of the soil solution was measured using a pH meter from INESA Scientific Instrument, Shanghai, China. Conductivity was determined following the method for preparing a saturated slurry [28]. Soil organic matter content was assessed via the external heating method with potassium dichromate, which also evaluated soil fertility [29]. The levels of nitrogen, phosphorus, and potassium in the soil were quantified to reflect nutrient status. Total nitrogen content was determined using the Kjeldahl method, total phosphorus content was assessed with the NaHCO_3_ extraction technique, and total potassium content was measured using an inductively coupled plasma optical emission spectrometer (ICP-OES 6300) from Thermo Corporation, Waltham, MA, USA [30].

#### 2.3.2. Analysis of Maize Production

Maize was collected from the experimental plots, and the harvested crop was weighed and analyzed for moisture content. The maize yields were then used to assess the effects of various fertilizer application regimes. Yield was computed using the following Formula (1):

(1) $$Y=10000/S\times W\times \left(1-M\right)$$

where Y represents the yield (kg ha^−1^), S signifies the area of the experimental field (m^2^), W denotes the total mass of maize at harvest (kg), and M denotes the moisture content of maize (%).

#### 2.3.3. Analysis of Mycorrhizal Infestation

All roots were meticulously cleansed with deionized water, and subsamples of fresh roots were weighed for mycorrhizal assessments, utilizing the gridline intersection method [31].

#### 2.3.4. DNA Extraction, PCR Amplification and Sequence Analysis

DNA extraction was executed utilizing the EZNA Soil DNA Kit (Omega Bio-Tek, Norcross, Georgia, USA) in strict accordance with the manufacturer’s guidelines. The integrity of the DNA was evaluated through quantitative assessment and qualitative analysis via NanoDrop 2000 (Thermo Fisher Scientific, Wilmington, DE, USA) and 1% agarose gel electrophoresis, respectively, to scrutinize the concentration, quality, and purity of the isolated DNA. PCR amplification was conducted employing ds 338F and 806R primers specific to the bacterial V3–V4 region, and ITS1F and ITS2R primers tailored for the fungal ITS hypervariable region.

#### 2.3.5. Data Processing

Raw data were consolidated utilizing FLASH v1.2.11, followed by filtration with Trimmomatic v0.33 [32]. Chimeric sequences were discerned and excised through UCHIME version 8.1, and high-quality labels were procured [33]. USEARCH (version 10.0) aggregated the purified labels into operational taxonomic units (OTUs) at a 97% similarity threshold [34]. The OTUs were subjected to filtration when the re-advancement fell below 0.005%. Species annotation and taxonomic investigations were conducted via the Silva database (for bacteria) and the Unite database (for fungi), with the benchmark set at 0.8.

#### 2.3.6. Statistical Analysis

Soil and plant data were subjected to one-way analysis of variance (ANOVA) complemented by Duncan’s post hoc tests. Significant discrepancies among treatments were evaluated through the ANOVA procedure utilizing DPS v17.0 software at a significance level of *p* < 0.05, with distinctions denoted by varying letters. Serial data were contrasted employing R 4.3.1 software. In particular, alpha diversity was gauged by several fundamental metrics, including ACE, Chao1, Simpson, Shannon, and coverage, and was compared via Student’s *t*-test. Beta diversity was assessed through principal coordinates analysis based on the Bray–Bray–Curtis, Jaccard, weighted and unweighted uniform distance algorithms.

## 3. Results

### 3.1. Effect of Fertilization Practices on Soil Chemical Properties

**Figure 2:**
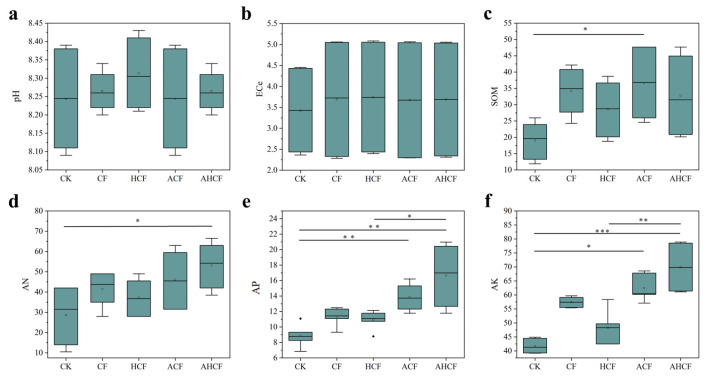
Changes in soil chemical properties of different fertilizer treatments (**a**): pH, (**b**): ECe, (**c**): SOM, (**d**): AN, (**e**): AP, (**f**): AK. Boxplots display median (central line), interquartile range (IQR, box edges), average (squares), and whiskers extending to 1.5× IQR. Black diamonds indicate outliers beyond the whisker line. Error bars denote the standard error of the mean. Asterisks indicate significant differences determined by Tukey’s HSD test (* *p* < 0.05; ** *p* < 0.01; *** *p* < 0.001).

The chemical properties of the soil under different fertilization treatments showed different patterns of change. The application of AMF-containing treatments significantly improved soil nutrient status compared to conventional fertilization (CK). In saline–alkali soil, the available potassium (AK) content increased by 53.5% (ACF: 60.37 vs. CK: 39.33 mg/kg) and 77.7% (AHCF: 61.35 vs. CK: 39.33 mg/kg) under ACF and AHCF treatments, respectively (*p* < 0.05) (Figure 2). Notably, AHCF-treated soil exhibited 39.6% higher AK than HCF treatment (61.35 vs. 44.17 mg/kg). Similarly, available phosphorus (AP) content showed a 33.9% increment in AHCF treatment relative to CK (20.50 vs. 15.50 mg/kg). While ammonium nitrogen (AN) was exclusively enhanced in AHCF treatment, achieving a 57.3% elevation over CK (64.17 vs. 40.83 mg/kg), soil organic matter (SOM) content demonstrated the most pronounced improvement under ACF treatment, with a 96.4% increase compared to CK (46.98 vs. 23.91 mg/kg). However, the treatments did not significantly impact the pH and ECe values of the saline–alkali soil, likely due to dynamic changes in water and salt content within the experimental field, which caused considerable fluctuations in the data. Detailed data can be found in Table A1. The application of AMF resulted in a significant elevation of the AK, AP, AN, and SOM contents within the saline–alkali soil.

### 3.2. Effect of Fertilization Practices on Maize Yield

**Figure 3:**
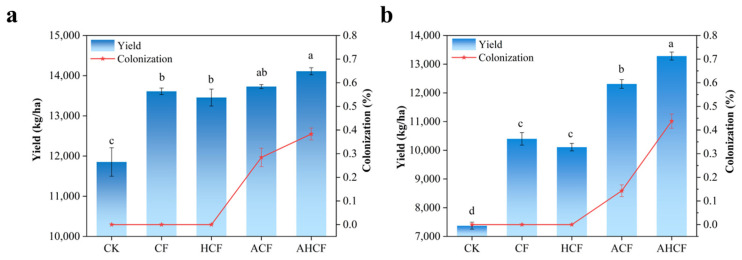
Effect of different fertilizer treatments on maize yield in Bei (**a**) and Xu (**b**). Bars represent the mean ± SE (*n* = 5 biological replicates). Error bars denote the standard error of the mean. Bars sharing the same lowercase letter are not significantly different (Tukey’s HSD test, *p* < 0.05). Letters assigned based on post hoc comparisons, with alphabetical order unrelated to treatment effect magnitude.

Maize yield exhibited treatment-dependent increases across both experimental sites. In Bei field, yield hierarchy followed CK (11,475 kg/ha) < HCF (13,695 kg/ha) < CF (13,260 kg/ha) < ACF (13,710 kg/ha) < AHCF (14,175 kg/ha), with AHCF yielding 23.5% higher than CK (Δ = 2700 kg/ha). This productivity enhancement correlated with a 16.1% increase in arbuscular mycorrhizal colonization rate in AHCF (31%) versus ACF (36%). Parallel trends were observed in Xu field, where AHCF treatment produced 13,125 kg/ha, representing an 81.2% yield improvement over CK (7245 kg/ha). Statistical analysis confirmed the significant yield superiority of AHCF treatment across both sites (*p* < 0.01). A comparison of Figure 3 reveals that the overall yield in experimental field Bei exceeds that in experimental field Xu; however, the impact of different fertilization methods on yield is more pronounced in experimental field Xu. In both fields, the mycorrhizal fungal infection rate is higher in the AHCF treatment group than in the ACF treatment group. As the mycorrhizal fungal infection rate increases, a corresponding rise in maize yield is observed.

### 3.3. Alpha Diversity of Soil Bacterial and Fungal Communities in Fertilization Treatments

**Figure 4:**
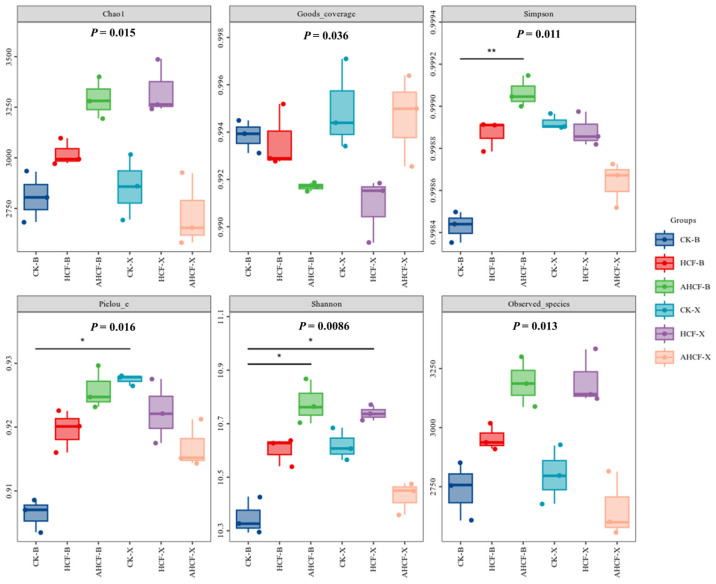
Soil bacterial α-diversity indices. Boxplots display median (central line), interquartile range (IQR, box edges), and whiskers extending to 1.5× IQR. Open circles denote outliers beyond whiskers. Error bars denote the standard error of the mean. Asterisks indicate significant differences determined by Tukey’s HSD test (* *p* < 0.05; ** *p* < 0.01).

**Figure 5:**
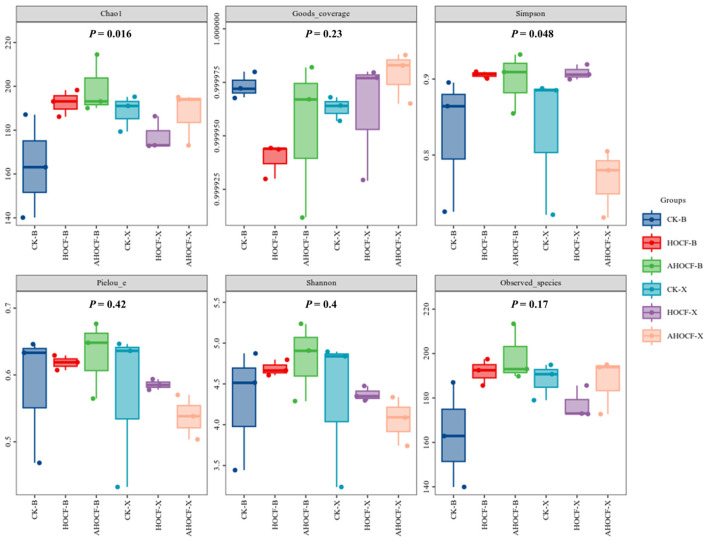
Soil fungal α-diversity indices. Boxplots display median (central line), interquartile range (IQR, box edges), and whiskers extending to 1.5× IQR. Open circles denote outliers beyond whiskers. Error bars denote the standard error of the mean. Asterisks indicate significant differences determined by Tukey’s HSD test.

Coverage refers to the extent of each sample library’s representation, with higher values indicating a greater likelihood of detecting sequences within the sample. This metric reflects the accuracy of sequencing results in representing the actual microorganisms present. The coverage indices for both bacteria and fungi in Bei and Xu surpassed 0.96, signifying that the sequencing outcomes accurately depict the microbial realities within the soil samples (Figure 4). Elevated values of community richness indices, namely Chao1 and Observed species, indicate greater community richness. For bacterial communities, the Chao1 and Observed species indices of the HCF and AHCF treatment groups in Bei and Xu were higher than those of the CK treatment group, with the AHCF treatment group exhibiting the highest indices. No significant disparity in bacterial community abundance was observed between Bei and Xu. For the fungal community, the Chao1 and Observed species indices in all treatment groups at Bei were higher than those in the CK treatment group. The highest fungal abundance was noted in the AHCF treatment group, while no significant differences were observed among the treatment groups in Xu (Figure 5). Additionally, no significant variance in fungal community abundance was detected between the two locations.

We also elucidated the diversity of bacterial and fungal communities through the Shannon and Simpson indices. A higher Shannon index signifies greater microbial diversity within the sample, while an elevated Simpson index indicates enhanced species evenness. For bacterial communities, the Shannon and Simpson indices for the HCF and AHCF treatment groups surpassed those of the CK treatment group in both Bei and Xu, with the AHCF treatment group exhibiting the highest levels of bacterial community diversity and evenness, showing significant differences in Bei village (Figure 4, *p* < 0.05). Conversely, the bacterial Shannon and Simpson indices were lowest in the CK treatment group across both sites. Regarding the fungal community, no significant differences in Shannon and Simpson indices were observed among treatment groups at the two locations, although the fungal community’s Simpson indices were reduced in the AHCF treatment at Xu.

### 3.4. Beta Diversity of Soil Bacterial and Fungal Communities in Fertilization Treatments

**Figure 6:**
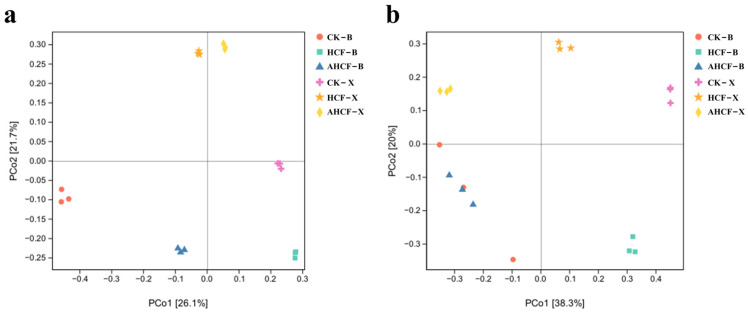
Principal coordinate analysis (PCoA) plot of bacterial (**a**) and fungal (**b**) community composition. Bars represent the mean ± SE (*n* = 3 biological replicates). PCoA ordination based on Bray–Cutis dissimilarity of bacterial OTUs. Axes labels include percentage variance explained.

PCoA analysis revealed that the presence or absence of compounded mycorrhizal fungi during fertilizer application significantly influenced the community composition of soil bacteria and fungi. For bacterial community structure, the first and second principal coordinates accounted for 26.1% and 21.7% of the variation among the six treatments, respectively (Figure 6a). Additionally, the bacterial communities in Bei and Xu were distinctly segregated along the second principal coordinate, with noticeable dispersion along the first principal coordinate among the treatments in Bei. Notably, the bacterial communities in the AHCF treatment group in Xu closely resembled the CK structure. For the fungal community structure, the first and second principal coordinates explained 38.3% and 20% of the variation, respectively (Figure 6b). Similarly, the fungal communities in Bei and Xu were clearly differentiated along the second principal coordinate. The fungal communities in the AHCF treatment group at Bei mirrored the CK structure, while those in the Xu treatment groups exhibited a marked divergence from the CK treatment group along the first principal coordinate axis.

### 3.5. Soil Bacterial and Fungal Community Composition and Relative Abundance in Fertilizer Treatments

**Figure 7:**
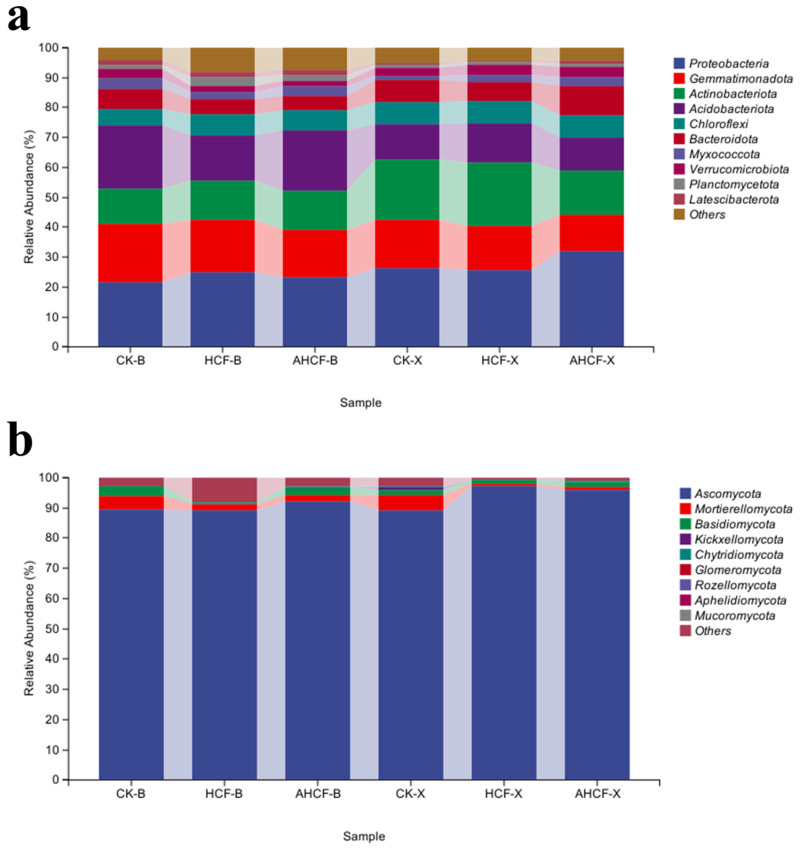
Relative abundance of bacterial (**a**) and fungal (**b**) communities at the phylum level. Stacked bars showing relative proportions of soil microbial communities.

The predominant six bacterial phyla identified were *Proteobacteria* (21.48–31.90%), *Gemmatimonadota* (12.19–19.07%), *Actinobacteriota* (11.59–21.40%), *Acidobacteriota* (11.26–21.42%), *Chloroflexi* (5.32~7.43%), and *Bacteroidota* (4.72~9.84%). Collectively, these six bacterial phyla constituted over 85% of the total sequence reads (Figure 7a).

The relative prevalence of the *Ascomycetes* and *Chlorobacteria* phyla was notably elevated in the Bei HCF and AHCF treatment groups compared to the CK group. In contrast, the relative prevalence of the *Bacillus* and *Acidobacteria* phyla was significantly higher in the CK treatment than in the HCF and AHCF treatment groups. In Xu, the relative prevalence of the *Ascomycetes* and *Actinobacteria* phyla was markedly higher in the HCF and AHCF treatment groups compared to Bei. However, the relative prevalence of the *Actinobacteria* phylum was comparatively lower in the HCF and AHCF treatment groups than in the CK group in Xu.

The predominant fungal phyla across all treatment groups were *Ascomycota*, *Mortierellomycota*, and *Basidiomycota*, constituting 89.12–97.17%, 0.72–5.78%, and 0.31–3.41% of all sequences, respectively (Figure 7b). Collectively, these three fungal phyla comprised over 95% of the high-quality sequences. In comparison to the CK treatment group, both AHCF treatments in Bei and Xu demonstrated a reduction in the relative abundance of the *Moltmannia* phylum and a corresponding increase in the relative abundance of the Ascomycota phylum, with the latter reaching its highest relative abundance in the AHCF treatment groups at both locations.

### 3.6. Analysis of Microbial Community Species Variation and Marker Species

**Figure 8:**
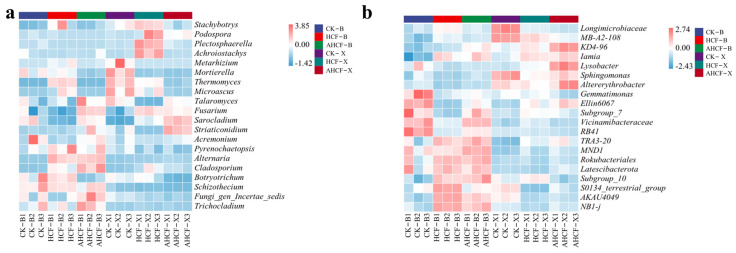
Heat map of species composition of bacterial (**a**) and fungal (**b**) communities. Color gradient from blue (low) to red (high) represents Z-score normalized relative abundance.

To further elucidate the variances in species composition among samples and present the trends in species abundance distribution across various samples, species composition analyses can be conducted using heat maps. We chose to utilize the abundance data of the top 20 genera by mean abundance to construct the heat map. In the CK treatment group in Bei Village, genera such as *Ellin6067*, *RB41*, *Gemmatimonas*, *Vicinamibacteraceae*, and *Subgroup_7* Bacteriophage, while genera like *TRA3-20*, *MND1*, and *Rokubacteriales* were more abundant in the HCF treatment group. Additionally, genera such as *Latescibacterota*, *Subgroup_10*, *S0134_terrestrial_group*, *AKAU4049*, and *NB1-j* bacteriophage exhibited higher abundance in the HCF treatment group (Figure 8a). In contrast, the AHCF treatment group showed an increased abundance of *Vicinamibacteraceae* based on the bacterial population observed in the HCF treatment group, along with a higher abundance of the *Subgroup_7* bacteriophage genus. The CK treatment group in Xu Village demonstrated a higher abundance of bacterial genera such as *Longimicrobiaceae*, *MB-A2-108*, and *Sphingomonas*, while genera *MB-A2-108* and *Iamia* were more abundant in the HCF treatment group. Moreover, genera *Iamia* and *Sphingomonas* were more prevalent in the AHCF treatment group, which also exhibited a higher abundance of bacteriophage genera such as Altererythrobacter, *KD4-96*, *Lysobacter*, and *Ellin6067*. Both sites displayed a decline in the abundance of Bacteroides spp. following the reduction of fertilizer application. Conversely, the combined application of AMF with reduced fertilizer not only altered the structure of Bacteroides spp. in the local area but also augmented their abundance.

*Trichocladium*, *Schizothecium*, *Botryotrichum*, and *Acremonium* fungal mycorrhizal genera were notably higher in the Bei CK treatment group. Conversely, *Schizothecium*, *Alternaria*, and *Pyrenochaetopsis* exhibited greater prevalence in the HCF treatment group, with *Botryotrichum* also demonstrating a heightened abundance. Furthermore, the AHCF treatment group displayed a more substantial abundance of *Fusarium*, *Talaromyces*, *Cladosporium FungigenIncertaesedis*, *Trichocladium*, and *Alternaria* fungal genera. In contrast, the CK treatment group was characterized by a higher abundance of *Thermomyces*, *Microascus*, *Metarhizium*, and *Mortierella* fungal genera, while the HCF treatment group was distinguished by a greater prevalence of *Stachybotrys*, *Podospora*, *Plectosphaerella*, and *Achroiostachys*. Additionally, the AHCF treatment group exhibited a more abundant presence of *Fusarium*, *Sarocladium*, and *Striaticonidium* (Figure 8b). This phenomenon was also observed among fungal genera exhibiting significant variability in both type and abundance across different treatments.

### 3.7. Differences in Metabolic Pathways of Bacterial and Fungal Communities in Fertilization Treatments

**Figure 9:**
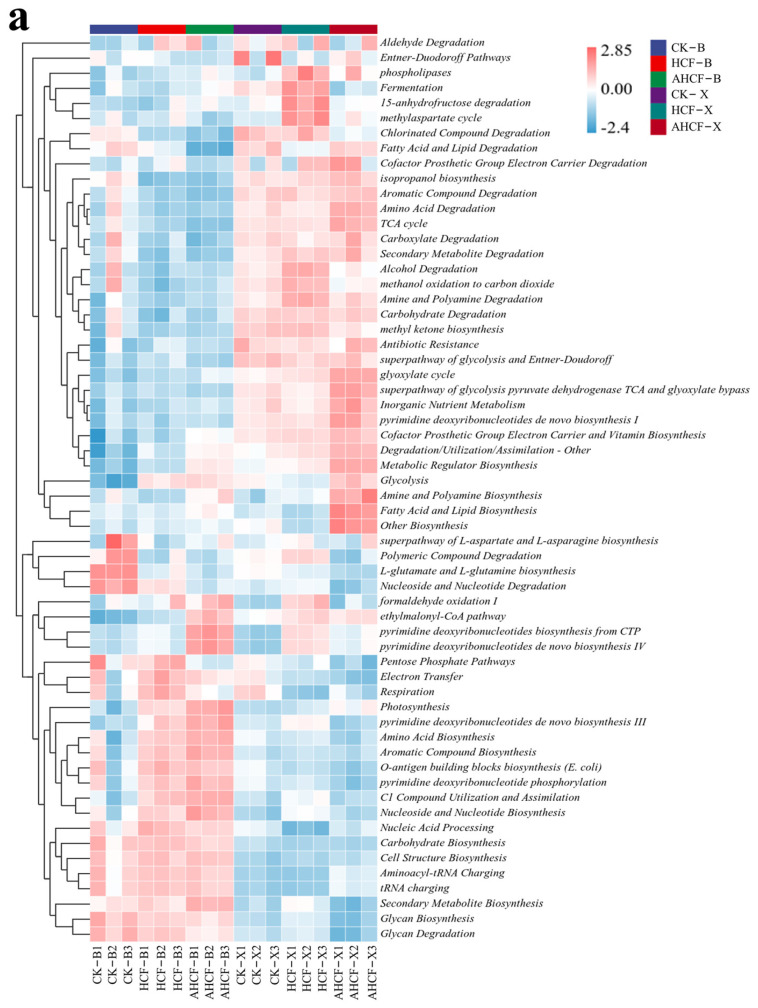

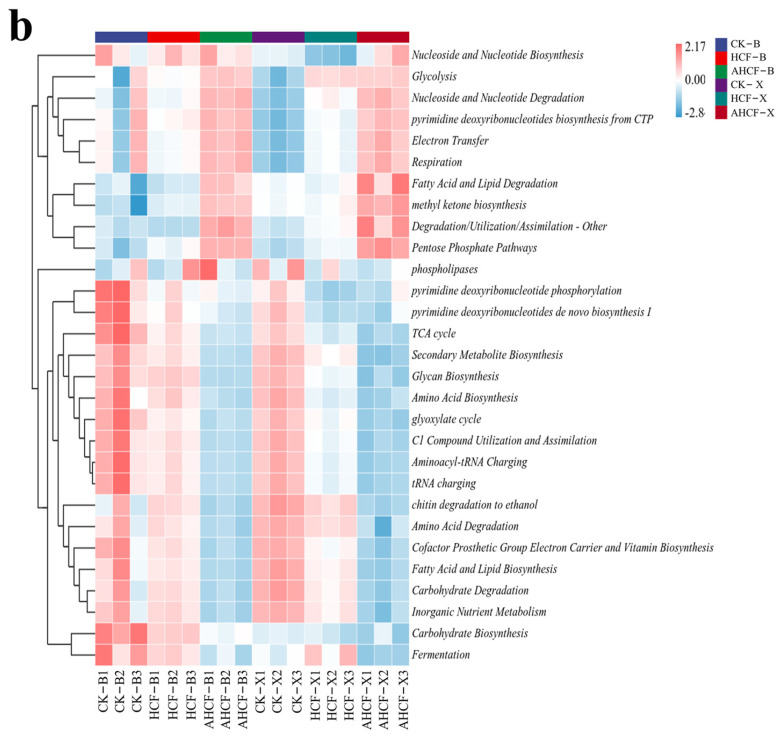
Heat map of metabolic pathway interactions in bacterial (**a**) and fungal (**b**) communities. Color gradient from blue (low) to red (high) represents Z-score normalized relative abundance.

To explore the correlation between various microbial genera and distinct treatments, the PICRUSt2 analysis was employed to forecast the functional potential inherent in the microbial communities, accompanied by the creation of a heat map. In the Bei HCF-treated group in comparison to the CK-treated group, encompassing the superpathways of *L-aspartate* and *glutamate biosynthesis*, as well as the degradation of *polymerization compounds*, *nucleosides*, and *nucleotides*; conversely, the AHCF treatment considerably augmented the pathways of *formaldehyde oxidation*, *ethylmalonyl cofactor metabolism*, and *pyrimidine deoxyribonucleotide biosynthesis* (Figure 9a). The HCF treatment in Xu, when juxtaposed with the CK-treated group, yielded significant outcomes in areas such as phospholipases, fermentation, 15-anhydrofructose degradation, and the methylaspartate cycle. Notably, the processes involving the biosynthesis of metabolic regulators, glycolysis, amine and polyamine biosynthesis, and fatty acid and lipid biosynthesis exhibited considerable significance, alongside other biosynthetic pathways.

It is evident that the composition of fungal metabolic pathways exhibited no marked alteration in the HCF-treated groups at Bei and Xu when juxtaposed with the CK-treated groups; however, the intensity was notably diminished (Figure 9b). Following the application of AMF, significant shifts were observed in the metabolic pathways of the fungal communities. The metabolic pathways of the fungal communities in the CK-treated and HCF-treated groups at the two sites were predominantly concentrated on metabolic pathways and cycles, biosynthesis and degradation, transformation and utilization, as well as fermentation and degradation. Conversely, the metabolic pathways of the fungal community in the AHCF-treated group encompassed nucleoside and nucleotide biosynthesis and degradation, sugar metabolic pathways, energy transfer and respiration, metabolism of fatty acids and lipids, and other related degradation and utilization processes.

### 3.8. Relationship Between Microbial Community Structure and Soil Chemical Properties

**Figure 10:**
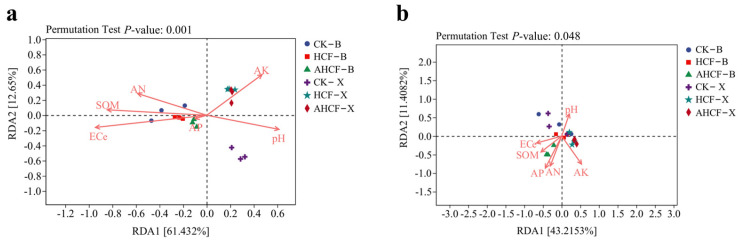
Redundancy analysis (RDA) of bacterial (**a**) and fungal (**b**) communities with soil chemical properties. Solid arrows indicate variables with VIF less than 5 and permutation test *p* < 0.05, and arrows are scaled by sqrt(r^2^) to prevent overplotting.

The relationship between soil chemical attributes and microbial community structure is elucidated through redundancy analysis (RDA), as depicted in Figure 10. The first two axes of the RDA explain 74.08% of the total variance within the soil bacterial community (Axis 1: 61.43%; Axis 2: 12.65%) (Figure 10a). Electrical conductivity (EC) shows the most significant correlation with the structure of the soil bacterial community, followed sequentially by soil organic matter (SOM), ammonium nitrogen (AN), pH, alkaline potassium (AK), and available phosphorus (AP). The first two axes of the RDA explain 54.63% of the total variance within the soil fungal community (Axis 1: 43.22%; Axis 2: 11.41%) (Figure 10b). Soil chemical attributes influence the soil fungal community composition in the following hierarchical order: AP > AN > AK > SOM > EC > pH.

## 4. Discussion

Maize yield is an important index for assessing the effects of chemical fertilizers (CF) and arbuscular mycorrhizal fungi (AMF). Figure 3a,b shows that the maize yield across all treatments in the two locations follows this order: CK < HCF < CF < ACF < AHCF. The difference in maize yield between CF and HCF in both locations was not significant, suggesting that nutrient utilization efficiency may be lower for the CF treatment [35]. It was observed that fertilizer application rates in the Loess Plateau of China reached 225–330 kg ha^−1^, significantly higher than the 97–141 kg ha^−1^ range. Moreover, high fertilizer inputs were found to reduce AMF diversity and shift community composition towards the dominance of a few weed taxa, which are less beneficial for crop growth [36]. Nutrients from chemical fertilizers are released more rapidly than those from organic fertilizers, potentially inhibiting AMF activity [37]. This explains the lower mycorrhizal infestation rate and maize yield in the ACF treatment group compared to the AHCF treatment group, a topic we will discuss further.

Previous studies indicated that the establishment of a symbiotic relationship between AMF and plants can effectively reduce soil pH and lower soil conductivity in saline soils [38]. However, this study found that AMF did not have a significant effect on the pH and electrical conductivity (ECe) values of local soils (Figure 2). The ECe values increased in the CF and HCF treatment groups in both experimental fields, likely due to fertilizer application, which raised the water-soluble salt content in the soil [39]. Both the ACF and AHCF treatment groups significantly increased the content of each nutrient element in the soil across the two fields, consistent with previous studies [40]. Estrada et al. demonstrated that mycorrhizal inoculation improved potassium accumulation while simultaneously reducing sodium levels in plants under salt stress [41]. Additionally, AMF can enhance host plant salt tolerance by increasing soil organic matter content in the root system [42]. Inoculation with AMF increased root vigor, average root diameter, total root volume, and root length in the 0.2–0.4 mm and 0.4–0.6 mm diameter ranges in maize, alleviating the damage caused by salt stress at salt concentrations of 0 to 2.0 g kg^−1^ [43].

The fertilizer reduction program significantly decreased the abundance of saline-dominant microbiota, converting it to another dominant microbiota. At the microbial community phylum level, the HCF group in the Bei experimental field increased the abundance of the bacterial phyla *Proteobacteria*, *Actinobacteriota*, and *Chloroflexi* compared to the group CK, while no significant difference was observed between the two treatment groups in the Xu experimental field (Figure 7a). Among these, *Proteobacteria* have a remarkable capacity for metabolism and degradation [44], whereas *Actinobacteriota* are eutrophic bacteria that play a key role in the soil carbon and nitrogen cycles [15]. In the Bei experimental field, the original bacterial populations *Gemmatimonas* and *Vicinamibacteraceae*—known for their role in organic matter decomposition—transformed into colonies of *Rokubacteriales* and *Latescibacterota*, which are involved in the synthesis of compounds and the cycling of carbon and nitrogen, following the application of chemical fertilizers (Figure 8a). This may be due to fertilizer application reducing the input of soil organic matter while increasing the input of nutrients such as carbon and nitrogen, leading to changes in organic matter-dependent microbial communities [45]. The Bei experimental field also saw an increase in the abundance of *Alternaria* and *Pyrenochaetopsis* fungi, while the Xu experimental field experienced an increase in the abundance of *Podospora*, *Plectosphaerella*, and *Achroiostachys* fungal mycorrhizal genera, significantly altering the structure of the fungal communities at both sites (Figure 8b). These findings are consistent with previous research [46].

The combination of fertilizer reduction and arbuscular mycorrhizal fungi (AMF) application led to further changes in the structure of saline microbial communities, which were more favorable for crop growth. The AHCF treatment increased the relative abundance of *Chloroflexi* and *Bacteroidota* while further decreasing the relative abundance of *Gemmatimonadota*. This change is presumably due to the competitive relationship between the *Bacteroidota* and *Gemmatimonadota*, which inhibited simultaneous increases in their relative abundances [47]. *Ascomycetes*, *Tephritobacteria*, and *Stramonium* are typical environmentally friendly saprophytic fungi that play an important role in soil health and function by enhancing soil nutrient effectiveness, influencing bacterial communities, and participating in soil carbon and nutrient transformations [48]. The changes in the structure of these microbial communities induced by AMF inoculation enhanced maize salinity tolerance, thereby improving its yield in saline–sodic soils. An increased abundance of the *Vicinamibacteraceae* bacterial genus was also observed in the Bei experimental field, alongside a further increase in the abundance of *Cladosporium*, *Fusarium*, and *Talaromyces* fungal genera within the AHCF treatment group (Figure 8). *Vicinamibacteraceae* can tolerate a wider pH range and up to 1% NaCl concentration [49]. As soil saprophytic fungi, *Cladosporium* and *Trichocladium* contribute to the decomposition of organic matter, promote soil nutrient cycling, and enhance soil fertility [50]; they can produce various enzymes, such as *cellulase* and *ligninase*, which aid in the decomposition of plant residues. Therefore, the application of AMF can effectively improve the soil environment and promote plant growth under saline and alkaline conditions, aligning with the results of higher crop yields in the AMF-containing treatment group shown in Figure 3.

Fertilizer reduction and the mixing of AMF were found to significantly alter the metabolic pathways of saline microorganisms, particularly fungal communities (Figure 9). The HCF-treated group increased the expression of metabolic pathways, such as nutrient cycling and electron transfer, consistent with previous studies [51]. In contrast, the AHCF-treated group showed increased activity in formaldehyde oxidation, the ethylmalonic coenzyme pathway, pyrimidine deoxyribonucleotide biosynthesis, gluconeogenic pathways, energy transfer and respiration, as well as metabolic pathways for fatty acids and lipids. This suggests that AMF acquire sugars from the plant root system and produce energy through sugar metabolism and respiration, while also converting nutrients from the soil into plant-available forms [52]. Nucleotide and lipid biosynthesis and degradation support cell division and structural integrity in fungi, while degradation and assimilation processes contribute to soil fertility and enhance the quality of the environment in which plants grow [53]. These metabolic processes not only contribute to the growth and reproduction of AMF but also enhance their supportive role for plants, thereby promoting soil health and plant growth [54].

The results of RDA analysis (Figure 10a) indicated that ECe, AN, SOM and pH were the primary drivers of bacterial communities in the soil. Previous scholars Nemergut et al. [55] found that AN can drive changes in soil bacterial communities. In addition, Bahram et al. [56] also found pH and AN to be important factors controlling microbial community aggregation. Furthermore, SOM has been shown to be a major driver in regulating microbial community composition [57], while the main drivers of the fungal community in this study were AN, pH, and AP (Figure 10b). Soil nutrients can indirectly affect fungal community composition by influencing changes in the aboveground parts of plants [58]. Nutrient levels were the primary parameters affecting fungal community composition. These results confirm that microbial community structure is most closely related to soil organic matter characteristics (e.g., SOM, nutrient type, and soil fertility), as soil carbon (C) and nitrogen (N) serve as the primary energy sources and constituent materials for fungi, influencing their spread by regulating metabolism [59]. Inoculation with mycorrhizal fungi can effectively increase AK, AN, AP, and SOM content in the soil, further enhancing the proliferation of fungal communities.

## 5. Conclusions

In this study, the combination of chemical fertilizer reduction and AMF fertilization was employed to improve the microenvironment of saline–sodic soil, thereby enhancing maize yield. It was found that reducing chemical fertilizer altered the microbial community and metabolic pathways in the soil; however, when combined with AMF, the negative effects were mitigated, further enhancing the abundance of saline-resistant microbial communities and promoting efficient nutrient utilization by plants. In saline–sodic soils, AMF primarily accumulated soil organic matter, nitrogen, phosphorus, and other nutrient ions, thereby reducing the toxicity of saline–sodic conditions and improving the overall soil microenvironment. The application of arbuscular mycorrhizal fungi (AMF) significantly modulates the rhizosphere microbial community structure of maize, promoting the development of a plant-growth-promoting microenvironment. Notably, the strategic integration of reduced chemical fertilizer input with AMF inoculation demonstrates synergistic effects in enhancing soil fertility and fundamentally restructuring microbial community composition through nutrient cycling optimization.

## Figures and Tables

**Table 1:** Initial basic physical and chemical properties of the soil at the study site.

Test Site	pH	Ece (ds/m)	SOM (mg/kg)	AN (mg/kg)	AP (mg/kg)	AK (mg/kg)
**BeiWuLao**	8.44	4.1 ± 0.25	25.42 ± 1.32	41.42 ± 2.11	9.22 ± 0.84	41.52 ± 0.84
**XuJiaZhen**	8.34	4.4 ± 0.41	15.76 ± 2.46	18.69 ± 2.43	9.43 ± 1.47	46.43 ± 0.55

## Data Availability

The original contributions presented in this study are included in the article. Further inquiries can be directed to the corresponding author.

## Abbreviations

The following abbreviations are used in this manuscript:

AMF	arbuscular mycorrhizal fungi
ECe	electrical conductivity of saturated soil extracts
SOM	soil organic matter
AN	available nitrogen
AK	available potassium
AP	available phosphorus
CK	conventional fertilization
CF	chemical fertilizer at 600 kg/ha
HCF	chemical fertilizer at 300 kg/ha
ACF	chemical fertilizer at 600 kg/ha and AMF at 157.5 kg/ha
AHCF	chemical fertilizer at 300 kg/ha and AMF at 157.5 kg/ha
B	BeiWuLao
X	XuJiaZhen

## Appendix A

**Table A1:** Soil chemical properties under various fertilization treatments.

	Plan	pH	ECe (ds/m)	SOM (mg/kg)	AN (mg/kg)	AP (mg/kg)	AK (mg/kg)
BeiWuLao	CK	8.34 ± 0.01 a	2.414 ± 0.21 ab	23.91 ± 2.06 d	40.83 ± 2.02 c	8.14 ± 1.23 d	39.33 ± 0.15 d
	CF	8.24 ± 0.01 b	2.348 ± 0.32 abc	39.41 ± 3.63 b	47.83 ± 2.02 b	12.08 ± 0.57 c	55.77 ± 0.55 b
	HCF	8.22 ± 0.01 b	2.427 ± 0.24 a	32.56 ± 0.83 c	45.50 ± 3.50 bc	11.73 ± 0.44 c	44.17 ± 2.89 c
	ACF	8.11 ± 0.02 c	2.301 ± 0.16 c	46.98 ± 1.19 a	59.50 ± 3.50 a	15.50 ± 0.64 b	60.37 ± 0.21 a
	AHCF	8.23 ± 0.02 b	2.333 ± 0.19 bc	43.54 ± 4.96 ab	64.17 ± 2.02 a	20.50 ± 0.45 a	61.35 ± 0.23 a
XuJianZhen	CK	8.43 ± 0.01 a	4.431 ± 0.11 b	14.19 ± 2.86 d	17.33 ± 3.81 c	9.55 ± 1.37 c	44.17 ± 0.95 e
	CF	8.44 ± 0.02 a	5.043 ± 0.22 a	26.20 ± 1.73 b	32.00 ± 3.00 b	10.61 ± 1.13 abc	59.07 ± 0.65 c
	HCF	8.41 ± 0.03 ab	5.055 ± 0.09 a	19.72 ± 1.31 c	29.17 ± 2.02 b	10.14 ± 1.17 bc	49.02 ± 0.38 d
	ACF	8.38 ± 0.02 b	5.046 ± 0.21 a	31.86 ± 2.66 a	32.67 ± 2.02 b	12.20 ± 0.36 ab	67.83 ± 0.75 b
	AHCF	8.30 ± 0.04 c	5.075 ± 0.14 a	28.32 ± 0.58 ab	38.67 ± 3.25 a	12.79 ± 1.06 a	78.50 ± 0.40 a

Note: Values are expressed as the mean ± SE (n = 3). One-way analysis of variance (ANOVA) followed by Duncan’s test (*p* < 0.05) was employed; different letters in the columns indicate significant differences between the various fertilization treatments.
